# Sensors Based on Plasmonic-Photonic Coupling in Metallic Photonic Crystals

**DOI:** 10.3390/s120912082

**Published:** 2012-09-03

**Authors:** Xinping Zhang, Shengfei Feng, Jian Zhang, Tianrui Zhai, Hongmei Liu, Zhaoguang Pang

**Affiliations:** 1 Institute of Information Photonics Technology and College of Applied Sciences, Beijing University of Technology, Beijing 100124, China; E-Mails: 07061127@emails.bjut.edu.cn (J.Z.); trzhai@bjut.edu.cn (T.Z.); hmliu@bjut.edu.cn (H.L.); pangzhaoguang@163.com (Z.P.); 2 Department of Physics, Capital Normal University, Beijing 100048, China; E-Mail: fengshengfei@emails.bjut.edu.cn

**Keywords:** plasmonic-photonic coupling, metallic photonic crystals, sensitivity enhancement, angle-resolved tuning, waveguide thinning

## Abstract

An optical sensor based on the coupling between the plasmonic and photonic resonance modes in metallic photonic crystals is investigated. Large-area metallic photonic crystals consisting of periodically arranged gold nanostructures with dimensions down to sub-100 nm are fabricated using solution-processible gold nanoparticles in combination with interference lithography or interference ablation, which introduces a variety of fabrication techniques for the construction of this kind of sensor device. Sensitivity of the plasmonic response of the gold nanostructures to the changes in the environmental refractive index is enhanced through the coupling between the narrow-band photonic resonance mode and the relatively broad-band plasmon resonance, which is recognized as a Fano-like effect and is utilized to explore sensors. Theoretical modeling shows the characterization and the optimization of the sensitivity of this kind of sensor device. Theoretical and experimental results are demonstrated for the approaches to improve the sensitivity of the sensor device.

## 1. Introduction

Localized surface plasmon resonance (LSPR) or particle plasmon resonance (PPR) results from a kind of collective oscillation of the free electrons in metallic nanostructures, which leads to strong scattering and absorption of light and is generally characterized by optical extinction spectroscopy. The optical response of PPR depends strongly not only on the dimensions or shapes of the nanostructures, but also on the environmental dielectric constant or refractive index. This lays the basis for the applications of metallic nanostructures in sensors. A number of sensors based on LSPR have been demonstrated [1–5]. However, the sensitivity of these sensors is distributed in a broad range, depending on the shape, the size, and the arrangement of the metallic nanostructures [6–10]. If the metallic nanostructures are arranged periodically into arrays or photonic crystal structures [11–17], new photophysics may be explored from the interaction between photonic resonance modes and PPR to develop sensors, implying possibilities to break through the limit of the sensitivity defined by PPR.

Combining waveguide grating structures (WGS) and metallic nanostructures, a kind of waveguide metallic photonic crystals (WMPC) is produced based on the coupling between the waveguide resonance mode [18] and PPR, which stands out as a promising candidate for new sensors. In the spectroscopic response, the narrow-band WGS resonance mode cuts into the broad-band PPR mode, leaving steep falling and rising edges on both sides of the WGS resonance mode. This Fano-like effect [19] in the photonic-plasmonic coupling actually amplifies the spectroscopic response of PPR to the environmental change in refractive index and thus enhances the sensitivity of the sensor device. This has been verified experimentally in the detection of specific bioreactions [20].

In this paper, we introduce new mechanisms that may be utilized to further enhance the sensitivity of the sensor by narrowing the spectrum of the coupled mode through thinning the waveguide layer or by steepening the spectral edge of the coupled mode through optimizing the angle of incidence.

## 2. Waveguide Metallic Photonic Crystals

### 2.1. Description of the WMPC Device

**Figure 1.**
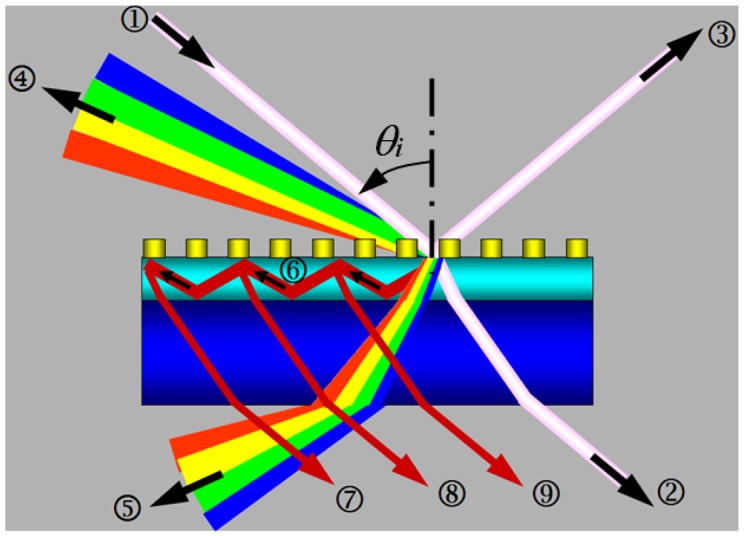
Photophysical processes in waveguide metallic photonic crystals.➀): incident light beam; (➁): transmitted beam; (➂): reflected beam; (➃): diffraction into the incidence space; (➄): diffraction into the transmission space; (➅): diffraction into the waveguide; (➆), (➇), (➈: secondary diffractions of the waveguide propagation mode by the grating.

Waveguided metallic photonic crystals consist of periodically arranged metallic nanostructures on top of a transparent waveguide layer, where gold is usually employed to construct the metallic nanograting structures and inorganic thin films are usually used as the waveguide. Indium tin oxide (ITO) [21] and zinc oxide (ZnO) [22] which have very little absorption in the visible spectral range and excellent stability in different environments have been used as the waveguide materials in our previous publications. As shown in Figure 1, the incident broad-band or white light beam ➀ is diffracted by the grating into multiple paths in addition to the transmitted beam ➁ and the reflected beam ➂: (1) the diffraction beam ➃ into the space of incidence with a condition of Λ(sin*θ_i_* + sin*θ_d1_*) = λ_1_, where Λ is the grating period, *θ*_i_ and *θ*_d1_ are the angles of incidence and diffraction, respectively, and λ_1_ is the wavelength of diffraction; (2) the diffraction beam ➄ into the space of transmission with a condition of Λ(sin*θ_i_* + *n*_2_ sin*θ_d2_*) = λ_2_, where *θ*_d2_ is the diffraction angle and *θ_d2_* < sin^−1^(*n*_3_/*n*_2_), *n_2_* and *n_3_* are the refractive indices of the waveguide and the substrate, respectively, and *λ_2_* is the diffracted wavelength. (3) the diffraction beam ➅ at a wavelength λ_3_ with a condition of Λ(sin*θ_i_* + *n*_2_ sin*θ_d3_*) = λ_3_, where *θ*_d3_ is the diffraction angle with *θ_d3_* < sin^−1^(*n*_3_/*n*_2_). Thus, the beam ➅) is totally reflected by both the top and the bottom interface, where we have considered that air has a smaller refractive index than the substrate and the condition for total reflection is more easily satisfied at the top interface. This propagation mode of the waveguide is diffracted further by the top grating structures into multiple beams (➆), (➇), (➈), ……) at the same angle of the direct transmission. For simplicity and clear demonstration of the principles by Figure 1, the diffractions of beam ➅ by the grating into the reflection space are not drawn in Figure 1. The destructive interference between beams ➆), (➇), (➈, …… (*E_S_* = Σ*_i_ E_i_*, i=➆), (➇), (➈)……) and the directly transmitted beam (➁ leads to the narrow-band modulation of the transmission spectrum. This is defined as the waveguide resonance mode of the waveguide grating structures and is observed as narrow-band dips in the transmission spectrum or narrow-band peaks in the extinction spectrum in waveguide dielectric grating structures. The corresponding physics have been described in reference [18].

However, if metallic materials are used to fabricate the grating structures, for example, gold nanolines are fabricated to construct the grating structures and the incident light is polarized perpendicular to the grating lines (TM polarization), strong LSPR or PPR may be excited. Thus, the incident light is strongly scattered and absorbed by the gold nanolines. This leads to significant reduction in the intensity of directly transmitted light beam and significantly enhanced propagation light intensity in the waveguide. Thus, the intensity of the diffracted light beams ➆), (➇), (➈), …… is much enhanced, which should in principle be observed as enhanced transmission or reduced extinction within a spectrum defined by the waveguide propagation mode. However, the destructive interference between beam (➆), (➇), (➈), …… and beam (➁) still exists, which results in the reduced intensity within the spectrum of waveguide resonance mode. Nevertheless, the total intensity of the diffracted beams is much higher than the intensity of beam (➁ and strong enhancement can be observed in the transmission spectrum. It should be noted that much broader-band enhancement in the propagation mode of the waveguide will be observed than that of the resonance mode of the waveguided dielectric grating structures. This is because the plasmonic scattering by the metallic nanostructures into the waveguide has much larger angular distribution than the diffraction by the grating.

If the light is polarized parallel to the grating lines, no particle plasmon resonance is excited. Both the scattered and the transmitted light intensity will be reduced due to the absorption by the metal. The intensity of waveguide propagation mode will not be enhanced. Narrow-band reduction in the transmission spectrum will be observed due to the destructive interference, as described above.

### 2.2. Optical Response of WMPCs

**Figure 2.**
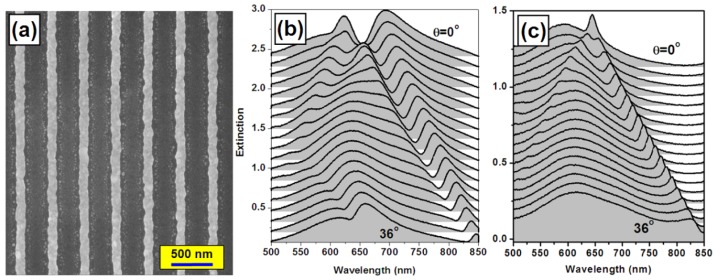
(**a**) SEM image of a WMPC device; (**b,c**): angle-resolved tuning properties of the optical extinction spectrum of the WMPC device for TM and TE polarization, respectively.

As has been discussed in Section 2.1, for metallic nanolines only TM-polarized light may excite PPR and induce the coupling between the waveguide and particle plasmon resonance modes. For TE polarization, PPR cannot be excited and only waveguide resonance mode can be observed, which appears as narrow-band extinction peaks. Figure 2(a) shows the scanning electron microscopic (SEM) image of the waveguided gold nanoline grating, which has been fabricated using an annealing temperature of 260 °C after the colloidal gold nanoparticles are spin-coated onto the photoresisit grating. The gold-nanoline grating has a period of about 400 nm and it is fabricated on a layer of ITO with a thickness of about 200 nm. It should be noted that at an annealing temperature of 260 °C, the photoresist master grating was not remove [23].

Figure 2(b,c) shows the optical extinction spectra of the device shown in Figure 2(a) for TM and TE polarizations, where the angle of incidence is increased from 0 to 36 degrees. Based on the +1 and −1 orders of diffraction by the grating, the waveguide resonance mode is split into two branches as the angle of incidence is larger than 0. This is basis for the optimization of the sensitivity of the sensors using WMPC, as will be discussed in section 4.

As shown in Figure 2(b), the coupled mode between the waveguide resonance and PPR appears as a dip in the extinction spectrum, implying enhanced transmission within the corresponding band. The longer-wavelength branch of the coupled mode is tuned from 653 to 830 nm. Figure 2(c) shows the angle-resolved tuning properties of the waveguide resonance mode for TE-polarized incident light. The narrow-band extinction peak is tuned from 645 to 826 nm. However, the waveguide resonance mode is actually superimposed on a broad-band spectrum that is centered around 600 nm. Clearly, the narrow-band and the broad-band features interact with each other through a simple linear superimposition. The broad-band feature results from the PPR of the small gold nanoparticles that are doped and randomly distributed in the photoresist after the annealing process, which can be observed between the periodically arranged gold nanolines. They induced extinction of the light through PPR without contributing to the modulation on the waveguide resonance mode due to their random distribution. Thus, no coupling between the waveguide resonance and this portion of PPR can be observed.

### 2.3. Fabrication Techniques

**Figure 3.**
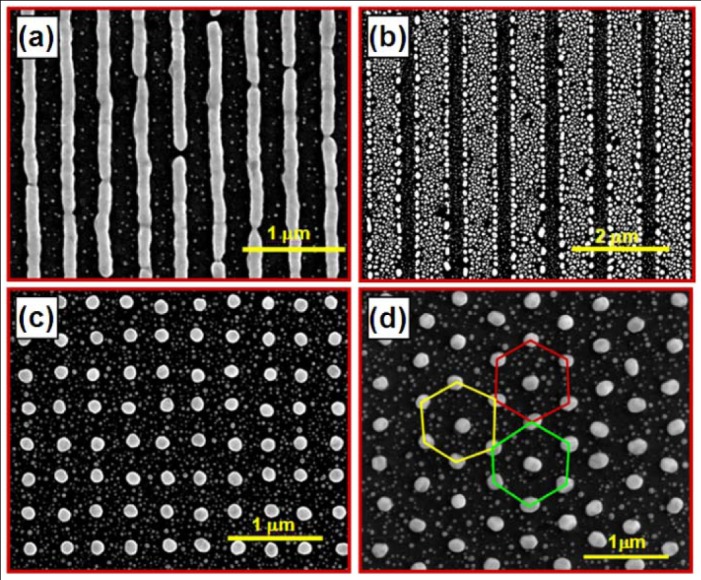
SEM images for different kinds of WMPC structures consisting of: (**a**) gold nanowires; (**b**) hybrid gold nanostructures; (**c**) square lattices of gold nanocylinders; (**d**) triangular lattices of gold nanocylinders.

A variety of techniques have been demonstrated to achieve metallic grating structures [24–26]. Solution-processible fabrication shows a number of promising features that are advantageous over the conventional approaches, as has been described in our previous publications [12,27]. Based on the colloidal gold nanoparticles that have been synthesized chemically, interference lithography and interference ablation [28] have been demonstrated for large-area nanopatterning of gold. In interference lithography, the interference pattern of UV laser beams is recorded by the spin-coated thin film of photoresist, producing a photoresist master grating in the first stage. Then the colloidal gold nanoparticles are spin-coated onto the grating structures. Based on the mechanisms of the natural confinement by the grating grooves and the surface energy modulation by the photoresist and the substrate, most of the gold nanoparticles are found in the grating grooves. In the final stage, the sample is annealed at a temperature above 200 °C, so that the ligands covering the gold nanoparticles are sublimated and the gold nanoparticle become molten to join together. Gold nanostructures thus form in the grating grooves. The large surface energy of the molten gold provides a further confinement mechanism, where the smaller molten gold nanoparticles tend to aggregate to larger ones. Figure 3 shows different WMPC structures that have been fabricated using interference lithography and the solution processible gold nanoparticles, which include one dimensional structures consisting of Figure 3(a) gold nanowires or Figure 3(b) randomly distributed gold nano-particles arranged into nanolines and two dimensional structures consisting of Figure 3(c) square and Figure 3(d) triangular lattices of gold nanocylinders.

**Figure 4.**
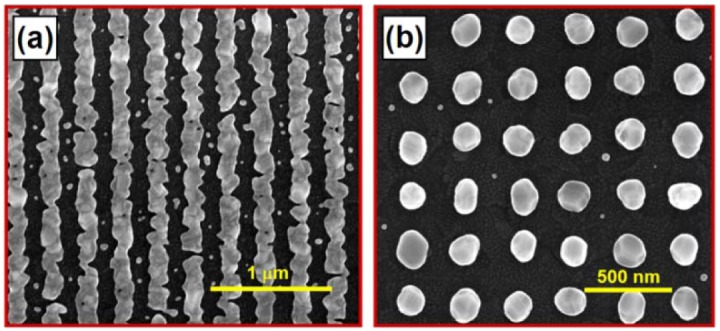
SEM images of the WMPC device fabricated by interference ablation, consisting of (**a**) gold nanolines and (**b**) square lattice of gold nanocylinders.

Interference ablation is a kind of direct writing technique and it is more efficient and more reproducible than interference lithography. The colloidal gold nanoparticles are first spin-coated onto the transparent substrate. The thin film of gold nanoparticles is then exposed to the interference pattern of UV laser beams. High pulse energy and short laser pulse lead to the instant removal of the gold nanoparticles within the bright fringes with nano-scale resolution, where strong absorption of the laser energy by the gold nanoparticles and very low thermal dissipation result in the immediate evaporation of the gold nanoparticles. The subsequent annealing process melts the gold nanoparticles and makes them aggregate into nanolines or nanocylinders. Figure 4 shows: (a) the one- and (b) the two-dimensional gold nanograting structures with a period of about 300 nm, which have been annealed at about 350 °C in a Muffle furnace.

Clearly, the gold nanolines fabricated using interference ablation are not as smooth and continuous as those fabricated using interference lithography. Using interference lithography, the confinement by the master gratings may not only define better shapes and smoother edges of the gold nanostructures, but also hold larger amount of gold in the photoresist grating grooves. However, using interference ablation, the amount of gold is fixed for each lattice site after the exposure process. This easily leads to breaking, irregular edges, small height/width ratio, and short-range homogeneity along the nanolines. This also explains why the two-dimensional structures show much better quality than the one-dimensional. Furthermore, it is understandable that the roughness of the gold nanostructures and the edge irregularity of the gold nanolines will broaden the spectrum of plasmon resonance, while reducing the strength of the coupled mode. This leads to the reduction in the amplitude and the contrast of the sensor signal.

A further advantage of the WMPCs fabricated using interference ablation for the application in sensors is that no template-removal is necessary. Very clean gold nanostructure can be obtained after the annealing process, as can be seen in Figure 4(b) and no additional materials like the remaining substance from the master grating will disturb the interaction between bio-molecules and the gold nanostructures. This favors very much the applications in sensors.

## 3. Sensors Using WMPCs

**Figure 5.**
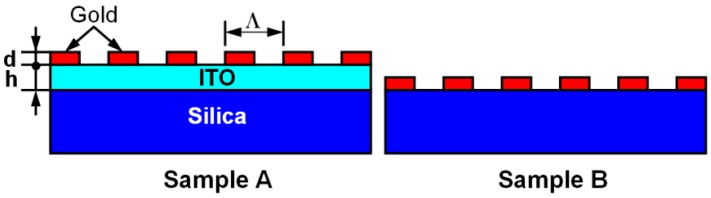
Schematic illustration of waveguide gold nano-line grating (**sample A**) and gold nano-line grating sitting on the substrate (**sample B**).

To characterize the sensors based on WMPCs, we make a comparison between two kinds of devices, as shown in Figure 5, where sample A is a kind of WMPC and sample B has no waveguide underneath the gold grating structures. We assume a grating period of Λ = 350 nm, a duty cycle of 0.5, and a modulation depth of about d = 20 nm. A 190-nm-thick ITO film (h = 190 nm) is used as the waveguide and it is assumed to have a constant refractive index of about 1.8 in the studied spectral range. The substrate is made of silica that has a constant refractive index of 1.45 in the studied spectral range. Simulations of the transmission spectrum through the samples A and B are performed using a commercial software (Gsolver) [29].

### 3.1. Response of Particle Plasmon Resonance to the Change in the Environmental Refractive Index

**Figure 6.**
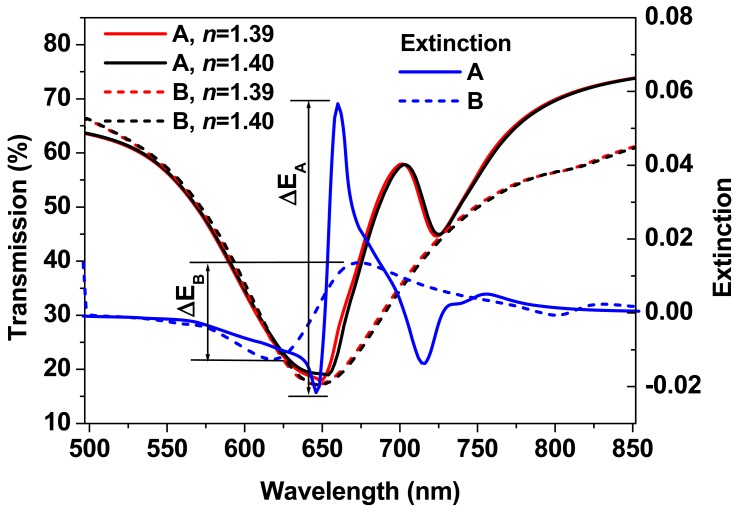
Simulation results showing the definition of the sensor signal and principles for the enhancement of the sensor signal through the coupling between the waveguide resonance mode and PPR in WMPCs.

The spectroscopic response of particle plasmon resonance is observed as a broad-band dip in the transmission spectrum due to the strong scattering and absorption of the light by the metallic nanostructures, which shifts to the red with increasing the environmental refractive index. Actually, how this spectral shift depends on the change of the environmental refractive index actually determines the sensitivity of sensors based on particle plasmon resonance. As shown in the simulation results in Figure 6, the dashed-red and dashed-black curves demonstrate the transmission spectrum of the gold-nanowire gratings without coupling with the waveguide resonance mode (sample A) for an environmental refractive index of 1.39 and 1.40, respectively. A spectral shift (Δλ) of only 1.8 nm is measured between these two curves, which is evaluated at the right edge of the transmission spectra. This leads to a sensitivity of Δλ/Δn = 180 nm/RIU (refractive index unity). Such sensitivity is quite small if compared with the conventional sensor devices. Due the broad-band properties of PPR, it is actually difficult to analyze the dip-to-dip spectral shift. Therefore, it is more convenient to characterize the sensor signal by the extinction spectrum, which is defined by:

(1) $$S_{B}(\lambda )=-\log\left[I_{B}(\lambda )/I_{B}^{0}(\lambda )\right]$$

where *I_B_*(*λ*) and $I_{B}^{0}(\lambda )$ correspond to the spectra after and before the change of the environment, thus, they correspond to n = 1.39 (the dashed red curve) and n = 1.40 (the dashed black curve) for the simulation results in Figure 6. The dashed blue curve is the calculated extinction spectrum *S_B_*(*λ*). The amplitude of *S_B_*(*λ*) is measured to be Δ*E_B_* = 0.0265 if using the peak-to-valley difference, implying a sensitivity of Δ*E_B_*/Δ*n* = 2.65 for a device of pure PPR.

**Figure 7.**
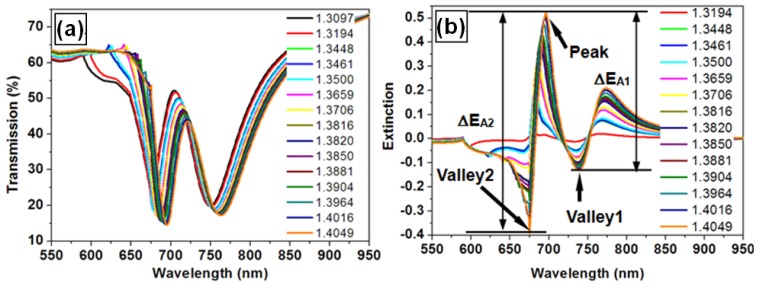

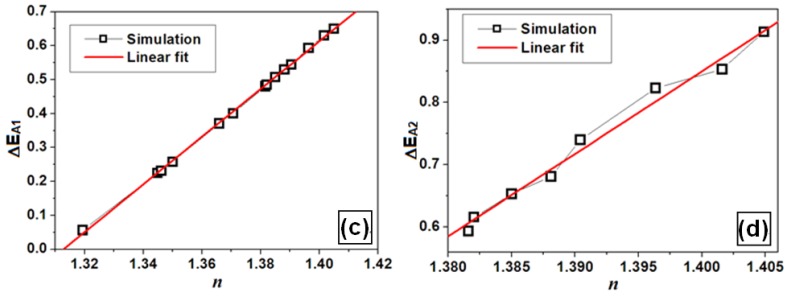
(**a**) The simulated transmission spectra at different environmental refractive indices; (**b**) The spectra of the sensor signal at different environmental refractive indices, where one peak and two valleys can be observed in the spectra and two values of the amplitude of the sensor signal can be calculated; (**c,d**): the plots of ΔE_A1_ and ΔE_A2_ as a function of the refractive index, respectively, and the corresponding linear fits to the calculated data.

Actually, the amplitude of the sensor signal depends linearly on the change in the refractive index. This is demonstrated by a series of simulations shown in Figure 7, where the gold nano-line grating has period of 450 nm, the ITO waveguide layer has a thickness of 190 nm and a refractive index of 1.8, and the silica substrate has a refractive index of 1.45. When the environmental refractive index is increased from 1.3097 to 1.4049, a series of transmission spectra can be simulated, as shown in Figure 7(a). Thus, the spectra of the sensor signals can be calculated using Equation (1), as shown in Figure 7(b), where one peak and two valleys can be observed. Consequently, two values (ΔE_A1_, ΔE_A2_) of the signal amplitude can be calculated. Figure 7(c,d) shows the simulation results of ΔE_A1_ and ΔE_A2_ as a function of the refractive index (*n*), where the open squares denote the simulation results and the red lines are the linear fits to the calculated values. Clearly, both the ΔE_A1_∼*n* and ΔE_A2_∼*n* relationships exhibit excellent linearity. In practice, the larger one between ΔE_A1_ and ΔE_A2_ is used as the amplitude of the sensor signal. However, the diffraction anomalies come into the simulation spectra and disturb the linearity of the ΔE_A2_∼*n* relationship, as shown in Figure 7(a,b,d).

### 3.2. Response Signals in Sensors Based on WMPC

For WMPCs, a narrow-band peak is observed in the broad-band dip in the transmission spectrum, which may be considered as a Fano-like effect and is observed at about 700 nm in the simulation results in Figure 6. The red $\left(I_{A}^{0}(\lambda )\right)$ and black (*I_A_*(*λ*)) solid curves in Figure 6 actually correspond to the transmission spectra of the WMPC device for an environmental refractive index of 1.39 and 1.4, respectively. Therefore, the sensor signal can be characterized by Equation (2) as:

(2) $$S_{A}(\lambda )=-\log\left[I_{A}(\lambda )/I_{A}^{0}(\lambda )\right]$$

The peaks in the solid black and red curves introduce much steeper rising and falling edges within the broad-band PPR spectrum, leading to much enhanced sensor signal, as can be measured using the solid-blue curve in Figure 6. The amplitude of *S_A_*(*λ*) is Δ*E_A_* = 0.0987 by the peak-to-valley difference, which is approximately 3.72 times as large as Δ*E_B_* and corresponds to a sensitivity of Δ*E_A_*/Δ*n* = 9.87. Thus, the coupling with the waveguide resonance mode actually plays a crucial role in the amplification of the sensitivity of the sensors based on PPR.

### 3.3. Enhancement of the Sensor Sensitivity through Photonic-Plasmonic Coupling

Amplification of the response sensitivity of PPR to the environmental refractive index modulation can be achieved through the coupling with the waveguide resonance mode, as have been demonstrated in Figure 6, where Δ*E_A_*/Δ*E_B_* ≈ 3.72. It is understandable that this amplification coefficient is dependent on the slope of the spectral edges of the coupled mode, which may be improved further by narrowing the bandwidth of the couple mode. Actually, the bandwidth of the coupled mode can be narrowed using thinner waveguide layer.

**Figure 8.**
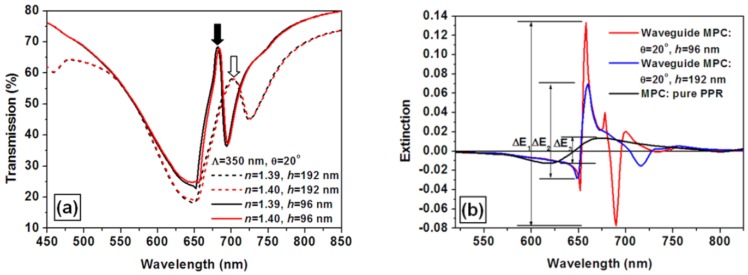
(**a**) Spectral narrowing of the coupled mode in the transmission spectrum through reducing the thickness of the waveguide; (**b**) The consequent further enhancement of the sensor signal through thinning the waveguide layer.

Figure 8(a) shows the simulation results of the transmission spectra for an incident angle of 20 degrees and for an environmental refractive index of 1.39 (black) and 1.40 (red) and for the structural parameters of the WMPC device of *Λ* = 350 nm, *h* = 96 nm (solid curves) and *h* = 192 nm (dashed curves). The reduction of the waveguide thickness from 192 nm to 96 nm leads to significant narrowing of the coupled mode, as can be verified by comparing the solid and the dashed peaks marked by the filled and empty arrows, respectively. This kind of narrowing results in further enhancement of the response sensitivity of the WMPC device.

Figure 8(b) shows a comparison between the sensor signals as the environmental refractive index increased from 1.39 to 1.40 for three different configurations of the sensor device: (1) waveguide MPCs with a waveguide thickness of 96 nm (the red curve); (2) waveguide MPCs with a waveguide thickness of 192 nm (the blue curve); (3) the MPCs without the waveguide structure (the black curve). The corresponding amplitudes of the sensor signals characterized by the peak-to-valley difference of the extinction spectra are Δ*E*_1_ = 0.211, Δ*E*_2_ = 0.0987, and Δ*E*_3_ = 0.0265, as depicted in Figure 8(b). Thus, the spectral narrowing of the couple mode leads to a further enhancement factor of Δ*E*_1_/Δ*E*_2_ = 2.14. The enhancement coefficient is then as large as Δ*E*_1_/Δ*E*_3_ ≈ 8.

## 4. Improvement of the Sensitivity through Optimizing the Incident Angle of Light

### 4.1. Basic Principles

**Figure 9.**
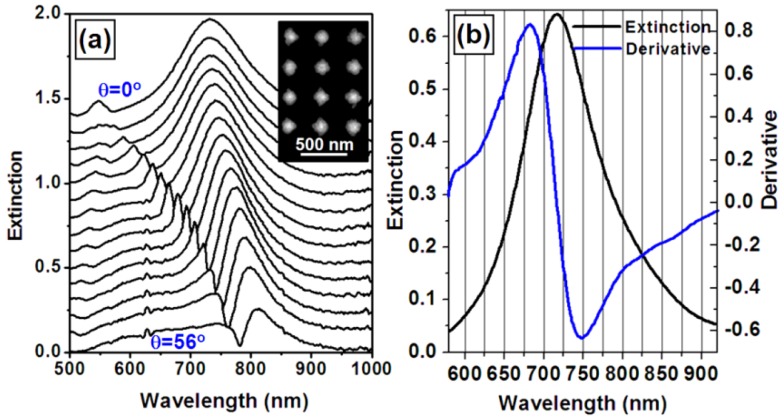
(**a**) Angle-resolved tuning properties of the optical extinction spectrum of the square-lattice WMPC shown in the inset; (**b**) The optical extinction spectrum (black curve) of the gold nanostructures without coupling with the waveguide resonance mode and its derivative (blue curve).

**Figure 10.**
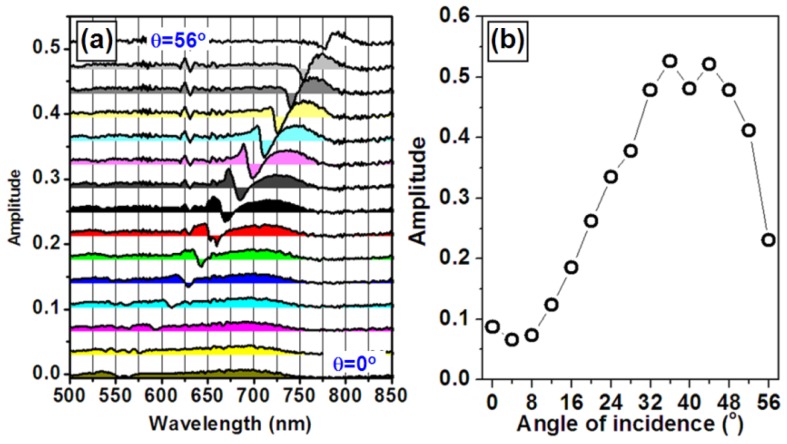
(**a**) The derivative curves of the optical extinction spectra in Figure 8(a); (**b**) The amplitude measured by the peak-to-valley difference of the derivative curves in Figure 9(a) as a function of the angle of incidence.

Another approach for improving the sensitivity of the WMPC sensors is to optimize the angle of incidence of the light onto the device. This is based on the principle that the waveguide resonance mode should be coupled with the spectrum of PPR at the position with the largest changing rate or the largest slope of the curve. Furthermore, the sensor signal due to the shift of the optical extinction spectrum is related directly to the derivative of the extinction spectrum. Then, the extremes of the derivative of the extinction spectrum may be used to guide the optimization of the sensitivity of the sensor device. Thus, the waveguide resonance mode may be tuned to overlap the spectral position of those extremes through tuning the incidence angle of the light. The black curves in Figure 9(a) are the measured optical extinction spectra of the WMPC device with the angle of incidence increased from 0 to 56 degrees in steps of 4 degree. The SEM image of the corresponding WMPC structures is shown in the inset. The black curve in Figure 9(b) shows the optical extinction spectrum of PPR of the gold nanostructures without coupling with the waveguide resonance, which is actually the optical extinction curve at *θ_i_* = 0 in Figure 9(a) and has very little overlap with the waveguide resonance mode. The blue curve in Figure 9(b) is the calculated results through differentiation operation on the black curves. The extremes of the blue curve can be found at about 680 nm and 750 nm. Thus, the sensitivity of the sensor device may be optimized by tuning the waveguide resonance mode to approximately 680 or 750 nm. Figure 10(a) shows the tuning of the derivative curves of the measured optical extinction spectra in Figure 9(a). If using the peak-to-valley difference to characterize the amplitude of the sensor signal, a curve for the optimization can be obtained in Figure 10(b), where the amplitude is plotted as a function of the angle of incidence. The peaks of the curve are found for incident angles in the range from 36 to 44 degrees, corresponding to the waveguide resonance mode ranging from 690 to 730 nm. This basically verifies our proposed principles.

### 4.2. Theoretical Simulations

**Figure 11.**
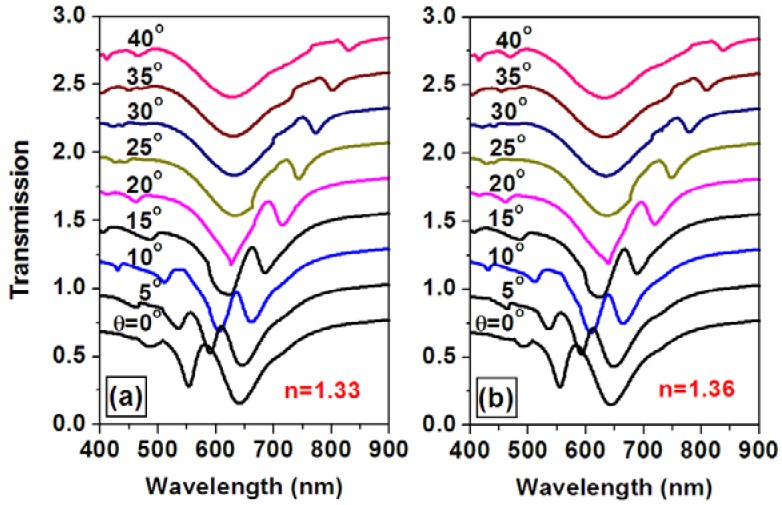
The simulation of the transmission spectra at different angles of incidence for an environmental refractive index of (**a**) 1.33 and (**b**) 1.36.

**Figure 12.**
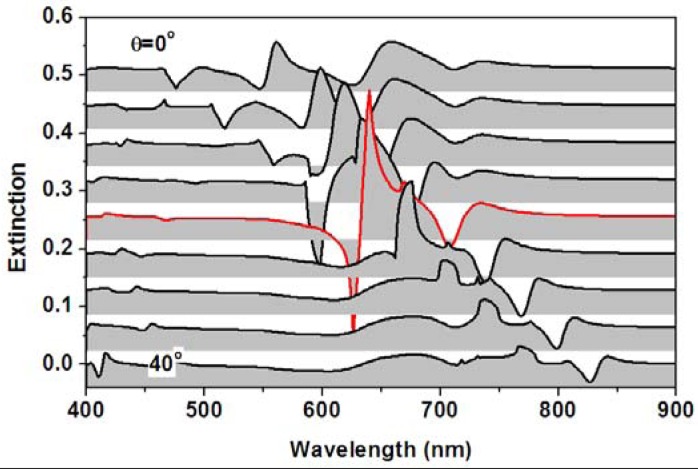
The extinction spectra at different angles of incidence (from 0 to 40 degrees in steps of 5 degrees) with the transmission spectra for n = 1.36 used as the signal and those for n = 1.33 as the blank.

Theoretical simulations were performed to demonstrate optimization of the sensor signal through tuning the angle of incidence or the spectral position of the overlap between the waveguide and plasmonic resonance mode. Figure 11 shows the angle-resolved tuning of the transmission spectrum through a WMPC device consisting of gold nanolines on top of the ITO waveguide for the TM polarization, where the incident angle is increased from 0 to 40 degrees. The spectrum of particle plasmon resonance is centered around 630 nm and the waveguide resonance mode in increased from 582 to 818 nm (the longer-wavelength branch). Figure 11(a,b) shows the simulation results for a refractive index of 1.33 and 1.36, respectively. If using the transmission spectrum at a refractive index of 1.33 as the blank and that at 1.36 as the signal to calculate the optical extinction spectrum, the changing of the sensor signal as a function of the angle of incidence may be obtained, as shown in Figure 12. Clearly, the amplitude characterized by the peak-to-valley difference in the sensor signal is strongly modified by changing the angle of incidence. The largest sensor signal can be observed at an incident angle about 20°.

### 4.3. Experimental Results

**Figure 13.**
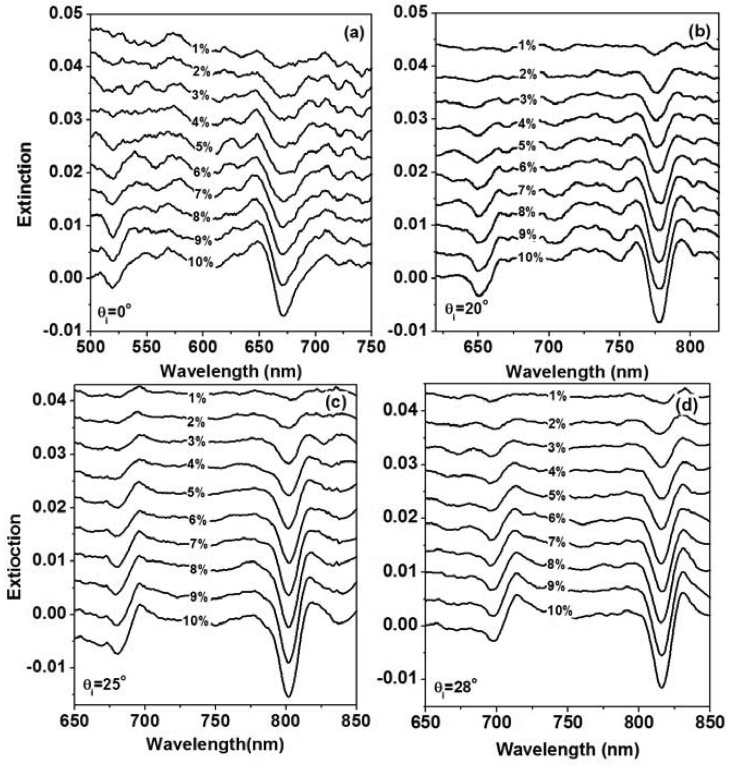
The measured sensor signals defined by the extinction spectra for glucose solutions with the concentrations changed from 1% to 10% with the transmission spectrum for pure water (a concentration of 0%) used as the blank for an incident angle of (**a**) 0; (**b**) 20 degrees; (**c**) 25 degrees; and (**d**) 28 degrees.

To verify the proposed principles experimentally, sensing experiments were performed on the glucose solution with the incident angle increased to 0 to 28 degrees and at each incident angle the concentration of the solution is increased from 0 to 10%, where the transmission spectrum at a concentration of 0% (pure water) is used as the blank to calculated the extinction spectrum (sensor signal). The sensor device in these experiments have been fabricated using interference lithography with subsequent spin-coating of the gold nanoparticles and the annealing processes, where the gold nanoline grating has a period of 400 nm and a modulation depth of about 100 nm. The width of the gold nanolines is about 150 nm. The gold nanoline grating is fabricated on an ITO waveguide layer that has a thickness of about 200 nm. Four sets of the measurement results are show in Figure 13, where Figure 13(a,d) shows the experimental data at an incident angle of 0, 20, 25, and 28 degrees, respectively. The evolution of the sensor signal with the concentration increased from 1% to 10% can be observed at each of the incident angle, where the sensor signal is centered at about 670, 780, 805, and 810 nm, respectively. Clearly, the amplitude of the sensor signal is different for different angles of incidence. In particular, at normal incidence (*θ_i_* = 0°), the bandwidth of the sensor signal is broader than those at other angles of incidence.

**Figure 14.**
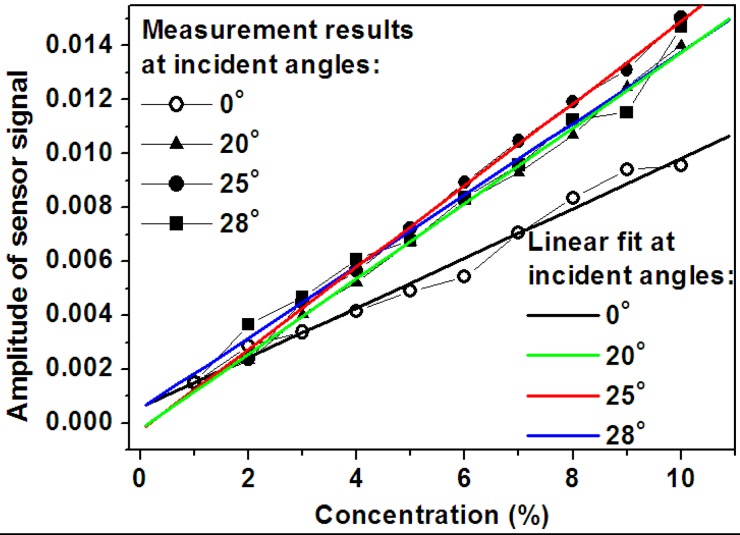
Amplitude of the sensor signal as a function of the concentration of the glucose solution at incident angles of 0, 20, 25 and 28 degrees. The solid lines show the corresponding linear fit to the measurement data.

Figure 14 summarizes the experimental results in Figure 13, where the amplitude of the sensor signal defined by the peak-to-valley difference is plotted as a function of the concentration of the solution sample. These data are fit using linear functions and the slope of each line actually measures the sensitivity of sensor at the corresponding angle of incidence. Clearly, at an incident angle of 25 degrees, the measurement data show a largest slope and thus the highest sensitivity, however, at normal incidence, the sensor shows its lowest sensitivity. Furthermore, at 20 and 28 degrees the sensitivity is also lower than that at 25 degrees, implying that an incident angle of 25 degrees is very close to the optimal value to obtain the highest sensitivity. Thus, tuning the angle of incidence is an effective approach to improve the sensitivity of the sensor device based on WMPCs. The linear fitting in Figure 14 was performed using the functions provided by the Origin software. Clearly, the fitting lines do not cross the (0,0) point. The reason for this is that the intensity of the light source may change slightly with time, which leads to a vertical shift of the extinction spectrum without changing the shape and the amplitude of the sensor signal.

## 5. Conclusions

Sensors based on waveguide metallic photonic crystals have been studied by demonstrating their basic physics, fabrication techniques, and principles for the enhancement of the sensitivity through the coupling between particle plasmon resonance and the photonic resonance mode in waveguided MPCs. Both theoretical and experimental results show effective routes for further enhancement of the sensitivity by adjusting the structural parameters of the MPCs and optimizing the incident angle of the light.
